# Comparison of revision rates and epidemiological data of a single total knee arthroplasty system of different designs (cruciate retaining, posterior stabilized, mobile bearing, and fixed bearing): a meta-analysis and systematic review of clinical trials and national arthroplasty registries

**DOI:** 10.1007/s00402-024-05286-6

**Published:** 2024-04-03

**Authors:** Anton Wagner, Ulrike Wittig, Lukas Leitner, Ines Vielgut, Georg Hauer, Reinhold Ortmaier, Andreas Leithner, Patrick Sadoghi

**Affiliations:** 1 Department of Orthopaedics and Trauma, Medical University of Graz, Auenbruggerplatz 5, 8036 Graz, Austria; 2 Ordensklinikum Linz, Barmherzige Schwestern, Seilerstätte 4, 4010 Linz, Austria

**Keywords:** Attune knee system, Total knee arthroplasty, Fixed bearing, Mobile bearing, Cruciate retaining, Posterior stabilized

## Abstract

**Background:**

This study aimed to meta-analyze epidemiological data, revision rates, and incidences of different designs of a single Total Knee Arthroplasty System and compare these factors across different countries.

**Methods:**

A systematic review was conducted on clinical studies and arthroplasty registries of ATTUNE TKA from 1999 to 2020. The main endpoints analyzed were revision rates and epidemiological data.

**Results:**

The average age of patients was 67.8 years, with a gender distribution of 60% female and 40% male. The pooled average BMI was 29.4 kg/m^2^. Eight clinical studies showed a pooled revision rate per 100 observed CY of 0.5 (n = 1343 cases). Cumulative revision rates after 1, 3, and 5 years varied among registries, with the Swiss registry having the highest revision data (after 5 years: 6.3%) and the American registry having the lowest revision data (after 5 years: 1.7%). A comparison of the revision rates of mobile bearing and fixed bearing (41,200 cases) as well as cruciate retaining and posterior stabilized (n = 123,361 cases) showed no significant advantage in the first 5 years after implantation.

**Conclusion:**

In conclusion, pooled data from 41,200 cases of TKA with a single Total Knee Arthroplasty System in two arthroplasty registries revealed that there was no significant difference in revision rates between the mobile bearing and fixed bearing design within the first 5 years after implantation. In addition, a comparison of the revision rates in n = 123,361 cases showed no significant advantage for cruciate retaining or posterior stabilized in the first 5 years after implantation.

## Introduction

Total knee arthroplasty (TKA) is considered the second most often performed endoprosthetic procedure after hip replacement. In Austria, registry data show a steady increase of performed procedures in the last decade. With 202 TKA procedures per 100,000 inhabitants annually, Austria is one of the leading countries in terms of nationwide coverage, when compared to other countries [1, 2].

The Attune TKA system (DePuy Synthes, Warsaw, Indiana) was introduced in 2013 and the suggested advantages were increased conformity of the inlay and femoral component, a gradually reducing radius, a newly designed posteriorly stabilized cam for gradual rollback for the posterior stabilized option, a broader range of sized in contrast to its predecessor, and an improved locking mechanism and additional antioxidant of the inlay. However, until today, no study has evaluated and compared all different options of the Attune TKA system with respect to survival using big data [3–10].

The aim of this study was (1) to meta-analyze pooled epidemiological data, revision rates, and incidences of different designs (cruciate retaining, posterior stabilized, mobile bearing, and fixed bearing) of the Attune TKA system and (2) to perform a comparison between different countries.

## Methods

### Search strategy

**Fig. 1:**
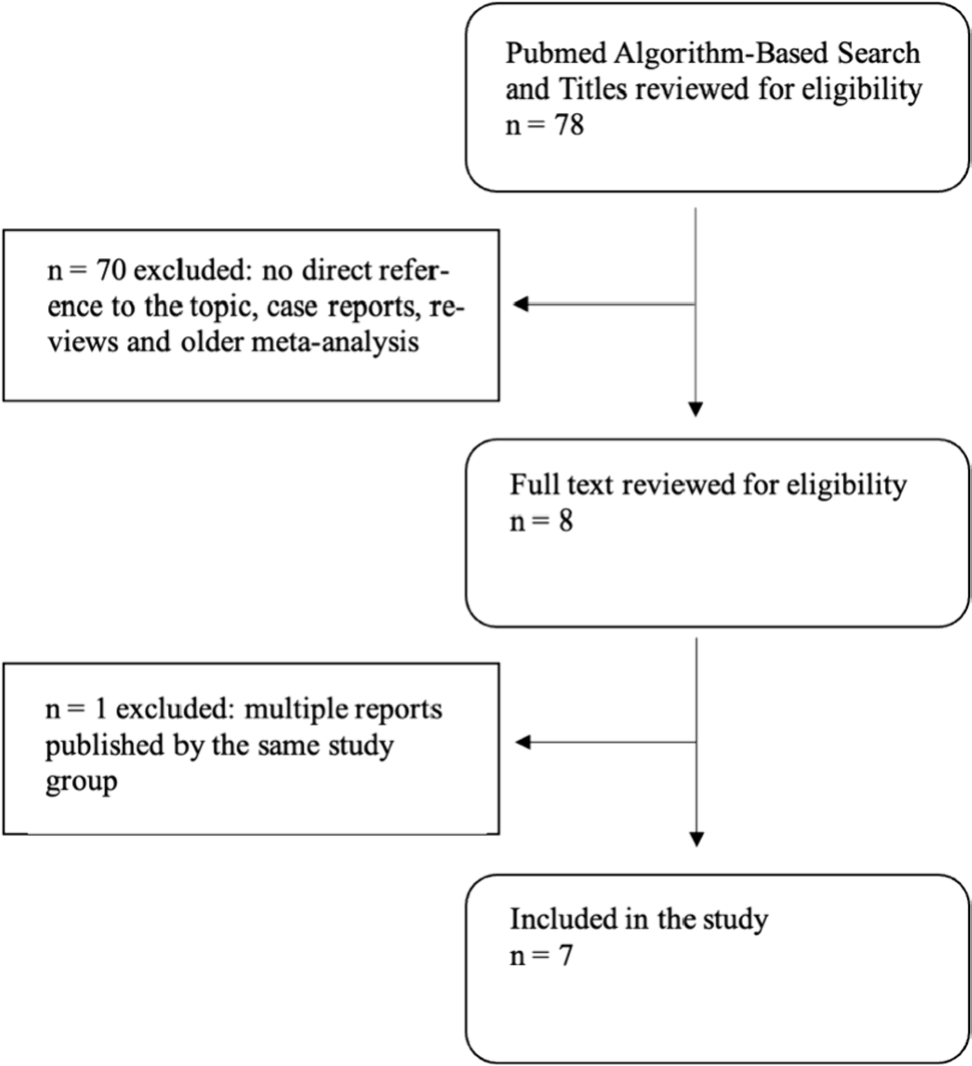
Flow chart of study identification using Pubmed

**Fig. 2:**
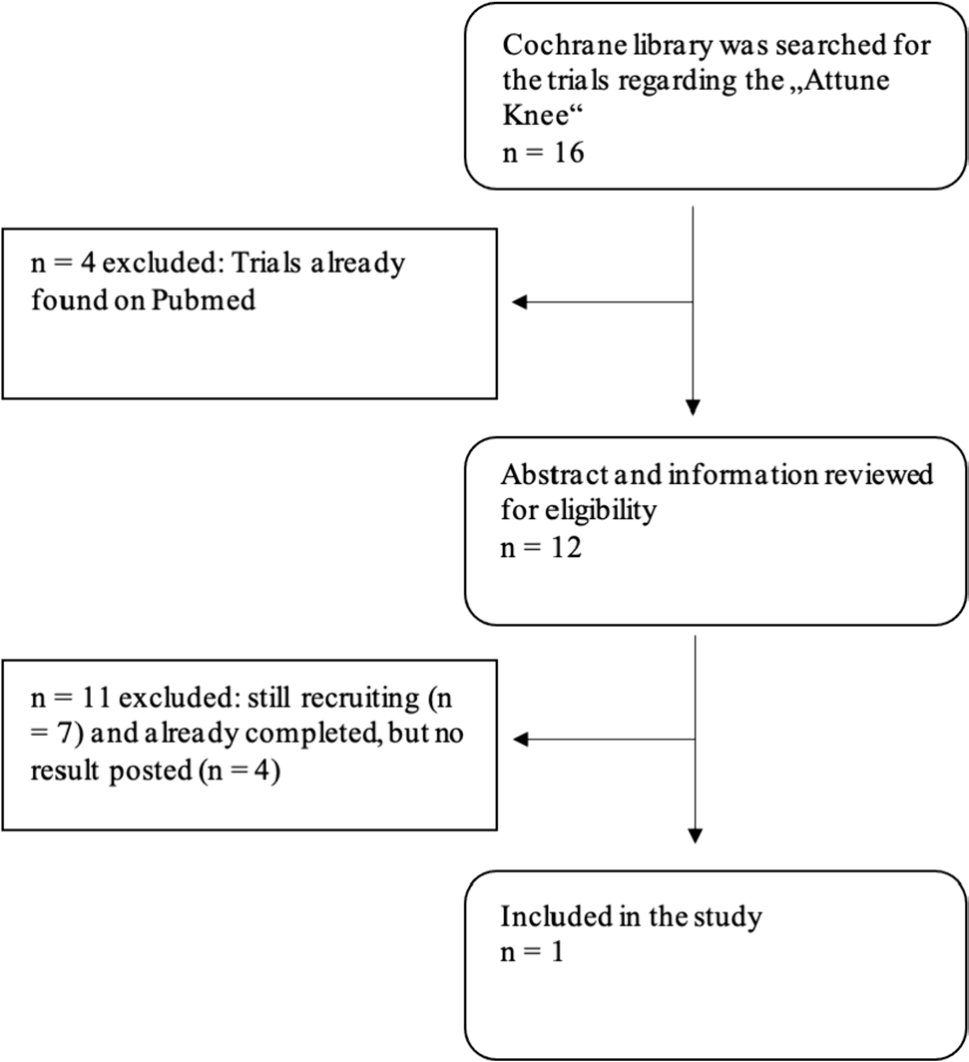
Flow chart of study identification using Cochrane

The Pubmed database and the Cochrane Controlled Trials Registry were systematically searched with the search terms (“Attune Knee”) or (“Attune Arthroplasty”) in May 2022. The search was conducted according to the PRISMA (Preferred Reporting Items for Systemic Reviews and Meta-analysis) guidelines [11]. The flow chart is shown in Fig. 1 for Pubmed and in Fig. 2 for Cochrane.

### Inclusion/exclusion pubmed and cochrane

Studies/Trials were considered suitable for qualitative analysis if the following criteria were provided: (1) The used implant must have been an Attune-Knee-System, (2) Follow-up time had to be two years or longer, (3) Number of people receiving Attune-Knee-Replacement, (4) The data had to be written in English or German and published in a peer-reviewed journal, (5) The studies had to be published between 2012 and 2022 (if there had been multiple reports from the same study group published in this period, the report with the longest follow up period was included).

Exclusion criteria were: (1) Case reports, (2) Reviews, (3) Older meta-analysis, (4) Imaging studies, (5) Cadaveric studies, (6) Studies with a follow up less than 2 months.

### Quality assessment

Pubmed studies and Cochrane trails: The level of evidence was determined using the Oxford Centre for Evidence-Based Medicine as illustrated in Table 1 [11]. All studies and trials were assessed for quality by 2 authors. (A.W. und U.W.) IRB approval was not necessary for this work according to our ethics committee.

**Table 1:** The level of evidence of included studies was determined using the Oxford Centre for Evidence-Based Medicine [11]

Study/trial	Level of evidence
Kaptein et al. (Pubmed)	II
Moorthy et al. (Pubmed)	III
Ruckenstuhl et al. (Pubmed)	I
Torino et al. (Pubmed)	I
Giaretta et al. (Pubmed)	I
Maniar et al. (Pubmed)	I
Vanitcharoenkul, Unnanuntana (Pubmed)	I
Ashraf et al. (Cochran)	I

### Data extraction

A full text data review of all Pubmed articles and Cochrane trials that met the inclusion criteria was performed. The two authors (A.W. and U.W.) independently extracted the relevant data from all eligible studies. Following criteria were assessed: (1) Authors, (2) Year of publication, (3) Duration of follow up time, (4) Country, (5) Study design, (6) Journal, (7) Baseline information of participants (median age, sex, BMI), (9) Type of prothesis, (10) Surgical technique, (11) Revision number (if published); Disagreements were resolved by consensus discussion between the two reviewers (A.W. und U.W.) and the senior author (P.S.).

### Arthroplasty registers

In May 2022, the Network of Orthopaedic Registries of Europe (NORE) [12] was checked for existing arthroplasty registries/annual reports. In addition, a free-hand search was performed using following terms: “(arthroplasty register) or (knee arthroplasty register)” at the searching engine “Pubmed” and “Google”. This method has been described in various studies before [13, 14]. Registers have had to meet following criteria to be included: (1) Reports had to be publicly available, (2) reports had to be written in German or in English, (3) the Attune-Knee-System had to be presented in the report, (4) and data had to be consistently reported for at least three consecutive years 2021–2020-2019 (date of retrieval 08.06.2022). Reports not available in German or English or reports with incomplete data were excluded from the study. Initially, 26 arthroplasty registers were found via the NORE Website and 5 via the free-hand search. Finally, 10 registers (Norwegian–NAR, Sweden–SKAR, German–EPRD, Swiss–SIRIS, England, Wales & Northern Ireland–NJR, Valdoltra (Slovenia)–VAR, America–AAOS, New Zealand–NZOA, Australia–AOANJRR and the Irish–NOCA) offered sufficient data and were included [17].

### Outcome measures

The outcome parameters were (1) revision rates after TKA with the Attune System, (2) a comparison of prosthesis types (mobile bearing versus fixed bearing as well as cruciate retaining versus fixed bearing), and (3) the description of epidemiological data (age, sex distribution, and BMI).

From a methodological standpoint, the calculation of p-values was not feasible in this study due to the utilization of real-life data rather than probabilities. To assess statistical significance, deviations from the mean were examined using a factor of three. This choice of a generous threshold was made to account for the numerous potential influencing factors present in the included datasets that are unrelated to the prosthesis itself, including surgeon expertise, patient characteristics, surgical techniques, and specific hospital circumstances. Deviations exceeding a threefold difference were considered significant as they could not be explained by these confounding variables as set in previous investigations using worldwide arthroplasty register data [12, 14]. The validity of this approach is supported by observations from the Swedish and Danish hip arthroplasty registries, where the revision rates of individual hospitals, when compared to the national average, fell within the threefold difference limit. Furthermore, the mean revision rates for individual implants did not exhibit variations exceeding a threefold difference across national registers [12].

Calculation of the component years (CY): To compare included studies in terms of included cases and time periods, the ratio "revision per 100 observed component years (CY)" was calculated. This allows us to compare different study data independent of their follow-up periods and the number of implanted prostheses. Component years (CY) is calculated as number of primary surgeries at follow-up multiplied by mean follow-up time. Larger cohorts and longer follow-up periods thus receive a higher weighting in comparison. The exact principle for the calculation of "revision per 100 observed component years (CY)" is the respective number of cases of revision surgery for any reason divided by the number of CY observed and multiplied by 100. A value of one revision per 100 observed CY corresponds to a 1% revision rate at 1 year and a 10% revision rate at 10 years of follow-up. This method has already been described and used in several studies [15–17].

## Results

### Age, gender, BMI (body mass index)

Seventy-eight manuscripts regarding the Attune TKA were revealed on Pubmed. After the search was completed, papers with no direct reference to the topic, case reports, reviews and older meta-analysis had been excluded (71 articles). The remaining publications (7 articles) were included into the study [18–24].

Twelve papers were found and reviewed for their eligibility to be included into the study in Cochrane. One article met the inclusion criteria and could be included into final analysis [25].

Of the ten arthroplasty registries including data regarding the Attune Knee System, two (German Arthroplasty Registry (EPRD) and National Joint Registry England, Wales, Northern Ireland, the Isle of Man, States of Guernsey (NJR)) provided epidemiological data (age and gender distribution). Registry data used was from the 2020 annual report, unless otherwise mentioned. Data on age, gender, and BMI is illustrated in Table 2.

**Table 2:** Data on age, gender, and body-mass-index (BMI) of clinical studies and arthroplasty registers providing data on the Attune total knee arthroplasty (TKA) system

Study/register	Median age in years	Gender M in%	Gender W in %	Median BMI
Kaptein et al. (Pubmed)	69	47.3	52.6	29
Moorthy et al. (Pubmed)	67.25	22	76	28
Ruckenstuhl et al. (Pubmed)	66.6	38.7	61.3	30.55
Ashraf et al. (Cochran)	64	47.6	52.4	29.9
EPRD (Registry)	70.5	37.1	62.9	No data
NJR (Registry)	69.6	43.3	56.7	No data

Three publications [18–20] published sufficient epidemiological data in the period from 2020 to 2022 included 205 patients who received primary TKA with the Attune TKA. Cochran's trail [25] included 64 patients who received primary Attune TKA in 2020. The two arthroplasty registries ((1) Germany–EPRD and (2) England, Wales, Northern Ireland, the Isle of Man, States of Guernsey–NJR) collectively included 45 101 Attune TKAs at of follow-up in 2020.

Pooled data of three clinical studies [18–20], the Cochrane trial [25] and the two knee registries (EPRD and NJR) showed an average age of 67.8 years (Range 64–70.5y) and a gender distribution of 60% female and 40% male at implantation (range men 22–44.6%; women 52.4–76%). The three clinical studies and the Cochrane trail showed a pooled average BMI of 29.4 kg/m^2^. (Range 28–30.6 kg/m^2^).

### Revision rates for attune total knee arthroplasty in clinical studies

The Cochrane trail: Ashraf et al. (n = 42) and 5 Pubmed studies: (1) Kaptein et al. [18] (n = 38), (2) Moorthy et al. [19] (n = 100), (3) Ruckenstuhl et al. [20] (n = 67), (4) Maniar et al. [22] (n = 72), (5) Vanitcharoenkul, Unnanuntana [23] (n = 54) had no revisions in Attune TKA.

Two Pubmed studies (1) Torino et al. [21] (n = 742) and (2) Giaretta et al. [24] (n = 228) had revisions in Attune-TKA. The mean follow-up period for the studies was 3.2 years (Range 2–5y). The Study by Giaretta et al. and the study by Torino et al. had the same median revisions/100 CY (0.7). All studies revealed 23 revisions. Cumulative data from all the clinical studies showed a total of 4346.1 observed CY. The pooled revision rate per 100 observed CY of all clinical studies was 0.53. This corresponds to a calculated revision rate of 5.3% within 10 years. Data is illustrated in Table 3

**Table 3:** Data on number of revisions and calculation of CY of the Attune total knee arthroplasty (TKA) system in Pubmed studies and Cochrane trails

Study/register	Mean Follow up-time (years)	Total number of follow-up arthroplasties	Total number of revisions (re)	Observed (CY) (n)	Rev / CY * 100
Kaptein et al (Pubmed)	2	38	0	76	0
Moorthy et al (Pubmed)	2	100	0	200	0
Ruckenstuhl et al. (Pubmed)	3.8	67	0	254.6	0
Torino et al (Pubmed)	3.5	742	18	2597	0.69
Giaretta et al (Pubmed)	3.16	228	5	720.5	0.69
Maniar et al (Pubmed)	2	72	0	144	0
Vanitcharoenkul, Unnanuntana (Pubmed)	5	54	0	270	0
Ashraf et al. (Cochran)	2	42	0	84	0
Total numbers all studies / pooled revision rate (total Revisions) per 100 observed CY (total CY)	**–**	1343	23	4346.1	0.53

### Revision rates for attune total knee arthroplasty in arthroplasty registries

Five registries (the German (EPRD), the Swiss (SIRIS), the American (AAOS), the Australia Registry (AOANJRR), and the England, Wales, Northern Ireland, Isle of Man, States of Guernsey (NJR)) published revision rates after 1, 3 and 5 years. The highest revision rates without differentiation between the different designs (MB/FB) or techniques (CR/FB) were published in Switzerland (SIRIS–after 5 years 6.3%) and the lowest were published in the American registry (after 5 years: 1.7%). Reasons for revisions were not published. Data on revision rates for all countries that published revision rates in Attune TKA is illustrated in Tables 4, 5, 6.

**Table 4:** Data on revision rates of the Attune total knee arthroplasty (TKA) system in worldwide arthroplasty registers. **a** Revision rates after 1, 3 and 5 years

National registry	PS/CR	FB/MB	Total number of follow-up arthroplasties	Revision Rate after 1 year	Revision Rate after 3 years	Revision Rate after 5 years
EPRD^1^	CR	FB	5802	1.6	3.1	3.6
	CR	MB	1417	1.4	2.8	3.2
	PS	FB	1362	2.5	4	5.9
	PS	MB	417	1	1.4	No data
SIRIS^2^	All	All	2954	1.7	5	6.3
AAOS^3^	PS	All	37,719	0.69	1.55	2.06
	CR	All	17,426	0.52	1.08	1.4
	All	All	55,145	0.61	1.32	1.73
NJR^4^	CR	FB	18,550	0.37	1.43	1.9
	CR	MB	3493	0.15	0.88	1.47
	PS	FB	10,159	0.45	1.62	2.48
AOANJRR^5^	CR	All	18,176	1	2.4	3.1
	PS	All	8840	0.9	2	2.7

^1^Cumulative revision rates from 2020

^2^Cumulative revision rates from 2020

^3^Cumulative revision rates from 2012–2019

^4^Cumulative revision rates from 2003–2020

^5^Cumulative revision rates from 1999–2020

**Table 5:** cumulative revision rates for a period and total numbers of revisions

National registry	Total number of follow-up arthroplasties	Year Data	Cumulative revision rate	Total number of Revisions
SKAR	114	2009–2018	2.14	
VAR	182	2002–2020		0
NZOA	12,075	1999–2020		193
NOCA	672	2014–2019	5.3

**Table 6:** cumulative revision rates after 1, 3 and 5 years

National registry	Revision rate after 1 year	Revision rate after 3 years	Revision rate after 5 years
EPRD^1^	1,625	2,825	3,175
SIRIS^2^	1,7	5	6,3
AAOS^3^	0,605	1,315	1,73
NJR^4^	0,323	1,31	1,95
AOANJRR^5^	0,95	2,2	2,9

^1^Cumulative revision rates from 2020

^2^Cumulative revision rates from 2020

^3^Cumulative revision rates from 2012–2019

^4^Cumulative revision rates from 2003–2020

^5^Cumulative revision rates from 1999–2020

### Fixed bearing (FB) and mobile bearing (MB)

A comparison of the revision rates between the fixed bearing and mobile bearing systems was carried out in the registries of Germany (EPRD), England, Wales, Northern Ireland (NJR) and New Zealand (NZOA).

### Fixed bearing (FB) and cruciate retaining (CR) or posterior stabilized (PS)

It was found that in 35,873 cases of fixed bearing implantation, there was no significant difference between cruciate retaining and posterior stabilized technique. Average revision rates for the combination fixed bearing/cruciate retaining (n = 24,352) in the German (EPRD) and England, Wales and Northern Ireland (NJR): after 5 years 2.8%. For the combination fixed bearing/posterior stabilized (n = 11,521): after 5 years 4.2%. Data is illustrated in Table 7.

**Table 7:** Data on revision rates of the Attune total knee arthroplasty (TKA) system in worldwide arthroplasty registers with respect to fixed bearing (fb)–cruciate retaining (cr) or posterior stabilized (ps) and mobile bearing (mb)–cruciate retaining (cr) or posterior stabilized (ps)

National registry	PS/CR	FB/MB	Total no. of follow-up arthroplasties	Revision rate after 1 year	Revision rate after 3 years	Revision rate after 5 years	No. of revisions total
EPRD^1^	CR	FB	5802	1.6	3.1	3.6	
	PS	FB	1362	2.5	4	5.9	
NJR^4^	CR	FB	18,550	0.37	1.43	1.9	
	PS	FB	10,159	0.45	1.62	2.48	
EPRD^1^ & NJR^4^	All	FB	35,873	1.23	2.54	3.47	
EPRD^1^ & NJR^4^	CR	FB	24,352	0.99	2.23	2.75	
EPRD^1^ & NJR^4^	PS	FB	11,521	1.48	2.81	4.19	
NZOA^6^	All	FB	4941				73
EPRD^1^	CR	MB	1417	1.4	2.8	3.2	
	PS	MB	417	1	1.4	No data	
NJR^4^	CR	MB	3493	0.15	0.88	1.47	
EPRD & NJR	All	MB	5327	0.85	1.69	2.34	
NZOA^6^	All	MB	5995				113

^1^Cumulative revision rates from 2020

^4^Cumulative revision rates from 2003–2020

^6^Total number of revisions from 1999–2020

### Mobile bearing (MB) and cruciate retaining (CR) or posterior stabilized (PS)

For the mobile bearing, cruciate retaining variant, higher revision rates were published in Germany (EPRD) than in England, Wales and Northern Ireland (NJR). (EPRD—after 5 years 3.2%) (NJR–CR/MB after 5 years 1.5%). Results for posterior stabilized-mobile bearing were only published in the German registry. Data is illustrated in Table 7

### Comparison mobile bearing (MB) and fixed bearing (FB)

A comparison of the design principles between mobile bearing and fixed bearing revealed no significant difference in the registers of Germany (EPRD) and England, Wales and Northern Ireland (NJR). For mobile bearing, the 2 registries (EPRD, NJR) with 5327 implanted prostheses showed average revision rates of 2.3% after 5 years. For Fixed Bearing, the 2 registries (EPRD, NJR) with 35,873 implanted prostheses showed a revision rates of 3.5% after 5 years. Data is illustrated in Table 7.

### Cruciate retaining (CR) and posterior stabilized (PS)

A comparison of the revision rates between the cruciate-retaining (CR) and the posterior- stabilization (PS) technique was carried out in the registries of Germany (EPRD), England, Wales, Northern Ireland (NJR), Australia (AOANJRR), America (AAOS), Sweden (SKAR) and New Zealand (NZOA).

### Cruciate retaining and fixed bearing versus mobile bearing

The registers from Germany (EPRD) and England, Wales and Northern Ireland (NJR) distinguished between FB and MB in the CR technique. The registries from America (AAOS), Australia (AOANJRR), Sweden (SKAR), and New Zealand (NZOA) did not distinguish between FB and MB in the CR technique. The German registry (EPRD) as well as the England, Wales and Northern Ireland registry (NJR) revealed no significant differences. Data is illustrated in Table 8.

**Table 8:** Data on revision rates of the Attune total knee arthroplasty (TKA) system in worldwide arthroplasty registers with respect to cruciate retaining (cr)–fixed bearing (fb) or mobile bearing (mb) and posterior stabilized (ps)–fixed bearing (fb) or mobile bearing (mb)

National registry	PS/CR	FB/MB	Total no. of follow-up arthroplasties	Revision rate after 1 year	Revision rate after 3 years	Revision rate after 5 years	No. of revisions total
EPRD^1^	CR	FB	5802	1.6	3.1	3.6	
	CR	MB	1417	1.4	2.8	3.2	
NJR^4^	CR	FB	18,550	0.37	1.43	1.9	
	CR	MB	3493	0.15	0.88	1.47	
AOANJRR^5^	CR	All	18,176	1	2.4	3.1	
AAOS^3^	CR	All	17,426	0.52	1.08	1.4	164
EPRD^1^, NJR^4^, AO-ANJRR^5^, AAOS^3^	CR	All	64,864	0.84	1.95	2.45	
NZOA^6^	CR	All	7424				123
SKAR^7^	CR	All	10				No data
EPRD^1^	PS	FB	1362	2.5	4	5.9	
	PS	MB	417	1	1.4	No data	
AAOS^3^	PS	All	37,719	0.69	1.55	2.06	
NJR^4^	PS	FB	10,159	0.45	1.62	2.48	
AOANJRR^5^	PS	All	8840	0.9	2	2.7	
EPRD^1^, NJR^4^, AO-ANJRR^5^, AAOS^3^	PS	All	58,497	1.11	2.11	3.29	
NZOA^6^	PS	All	4616				70

^1^Cumulative revision rates from 2020

^3^Cumulative revision rates from 2012–2019

^4^Cumulative revision rates from 2003–2020

^5^Cumulative revision rates from 1999–2020

^6^Total number of revisions 1999–2020

^7^Cumulative revision rates from 2009–2018

### Posterior stabilized and fixed bearing (FB) versus mobile bearing

The differentiation between the countries regarding posterior stabilization was difficult due to the very different published variants. Germany (EPRD) was the only country to differentiate between mobile bearing (MB) and fixed bearing (FB) in posterior stabilization (PS) implantation. Data is illustrated in Table 8.

### Comparison cruciate retaining (CR) and posterior stabilized (PS)

A comparison of the revision rates between cruciate retaining and posterior stabilized revealed no significant difference in the registers of Germany (EPRD), England, Wales and Northern Ireland (NJR), Australia (AOANJRR) and America (AAOS) for 123,361 cases. (CR/ALL after 5 years 2.5%, PS/ALL after 5 years 3.3%).

## Discussion

The aim of this study was to meta-analyze pooled epidemiological data, revision rates, and incidences of different designs (cruciate retaining, posterior stabilized, mobile bearing, and fixed bearing) of the Attune TKA system and to perform a comparison between different countries. The most important finding of this study was that there is no significant difference in revision rates between mobile bearing and fixed bearing and between cruciate retaining and posterior stabilized in the first five years after Attune Knee System implantation. The studies and registry data of the Attune Knee System were analyzed independently of each other in our study, as a direct comparison of the data is not possible due to a lack of differentiation between MB/FB and CR/PS in the studies.

The component years (CY) method is one way of comparing different studies with a different follow up time and a different number of revisions. The linear function of revision without differentiation of the reason represents a mathematical simplification of reality, which does not correspond to practice. For example, there is an increased risk of septic revision immediately after surgery and an increased risk of aseptic loosening later on. There are other methods to evaluate the data (Kaplan–Meier method, the cox model or the cumulative incidence) but these methods also have their own statistical errors as Ranstam et al. showed in his study [26, 27].

Eight studies that published data on Attune Knee showed a pooled revision rate per 100 observed CY of 0.53 A study by Hauer et al. [28], which used the same method to investigate unicompartmental knee arthroplasty without differentiation of the prosthesis company, showed slightly higher median revisions/100 CY of 0.8–1.16 [28]. Another study by Pabinger et al. with 168 included studies without differentiation with regard to a knee system showed a median revision rate in the studies of 0.7 revisions/100 CY. A limitation of our study and a possible reason for the lower results is certainly that only two studies [21, 24] that published data on Attune Knee TKA had revisions and that in most cases the studies were conducted by one department.

The large differences in revision rates in the different publishing department of a country’s arthroplasty registry show that there are better department that have a 2.5 times lower revision rate than those of the worst department. To assess statistical significance, deviations from the mean were examined using a factor of three. This approach is supported by various studies and by the Swedish and Danish registers [12, 26, 28].

A comparison between countries showed that Switzerland revealed the highest revisions after 5 years for Attune-TKA with 6,3%. It is difficult to compare the various knee prothesis between countries, as some registries do not published complete data (NJR) and others only publish cumulative revisions rates (SKAR, VAR, NZOA, NOCA), which makes a comparison with the annually published registers difficult. Studies show that the revision rate is influenced by various factors (surgeon, the hospital environment, the health care system, preventive factors) and it is therefore difficult to interpret the cause of high or low rates [29–31]. A detailed evaluation of the data would be needed to capture the cause of these differences. The large differences in the revision rates in the various registers should also be mentioned. For example, Switzerland published a cumulative revision rate after 5 years of 6.3%. America, on the other hand, only had a revision rate of 1.7% after 5 years. The fact that these large differences exist should prompt us to examine the data more closely and identify possible factors that could lead to a distortion of the data.

Our study showed no significant difference between mobile bearing or fixed bearing in the first 5 years after Attune implantation. A systematic literature analysis from Wittig et al. [32] without differentiation of a specific knee system in six registries revealed no significant difference in revision rates between mobile bearing or fixed bearing. It was revealed in this study that fixed bearing is more frequent used than mobile bearing. This is in line with our data.

A disadvantage of the mobile bearing variant described in the literature is dislocation [33, 34]. This could be the possible reason for the lower number of implantations of mobile bearing in the registries [13]. Other studies with a similar study design also showed no significant differences between mobile bearing and fixed bearing [3–5, 8, 35–37]. The choice between MB/FB does not seem to change the postoperative outcome [38]. Systematic reviews from Huang et al. and Capella et al. [38, 39] without differentiation of the specific prothesis also revealed that the theoretical advantages of mobile bearings (reduce the wear and the consequent loosening of the implant, improve ROM) over fixed bearings in terms of patient satisfaction, clinical, functional, and radiological outcome or medium and long-term survivorship revealed no significant improvement. This revealed that the results are similar even without differentiating a specific knee system [7, 39]. The comparison between cruciate retaining and posterior stabilized in the registries revealed no advantage in revision rates for any implantation technique in the first 5 years after Attune Knee implantation. This result was also shown in the previous studies without differentiation by the knee system [7, 40, 41]. The publications regarding CR/PS are very heterogeneous. Many surgeons tend to choose the type of prothesis based on their experience. Song et al. [42] published that for the decision of the prothesis type surgical indications and solid understanding should be applied instead of selecting either CR or PS prothesis. For example, CR TKA should not be feasible in following settings: posterior cruciate ligament insufficiency, severe deformity, and history of trauma or surgery or that the amount of distal femoral resection, femoral component size, and tibial slope are evident for the successful TKA [42]. An RCT by Li et al. [43] showed that the flexion angle was 11.07° and 2.99° higher for patients who received PS-TKA instead of CR-TKA. However, their study showed no statistical differences in knee society pain score, extension angel, 2- and 5-year knee society score, 2- and 5-year knee society function score and complications after TKA [43]. A systematic review by Kanna et al. showed that CR had a significantly better survival after 10 years (long time) than the PS variant [44]. The possible advantages of a variant (PS/CR) would have to be further investigated in clinical studies in order to confirm the advantages of a variant [7, 40, 41, 45]. As the reasons for revision were not published in the registers, it makes it difficult to interpret the overall revision rates for this implant without comparisons to other implants within the same sources of data.

The amount of implanted Attune prostheses in clinical studies is lower compared to the registers. All studies examined over the last 10 years had a total of 1343 followed up Attune knee arthroplasties. In comparison, the number of observed Attune TKAs in the annual report of the Swiss registry 2021 was 2954. This should be taken into account when reviewing the data. We would like to mention that clinical scores are missing in this meta-analysis, which is a limitation of our work. In addition to the revision rate, quality of life and patient satisfaction play a crucial role, which could also not be correlated to the different designs and techniques.

## Conclusion

In conclusion, pooled data from n = 41,200 cases of Attune TKA system in worldwide arthroplasty registers revealed that there is no significant advantage in revision rates for mobile bearing or fixed bearing in the first 5 years after implantation. In addition, a comparison of the revision rates in n = 123,361 cases also showed no significant advantage for cruciate retaining or posterior stabilized in the first 5 years after implantation. Eight studies of the Attune Knee System showed a calculated revision rate (Revisions/100CY) of 5.3% within the first 10 years after Attune-Knee implantation.
